# Improved High-Quality Draft Genome Sequence of *Pseudomonas fluorescens* KENGFT3

**DOI:** 10.1128/genomeA.00428-16

**Published:** 2016-05-26

**Authors:** Jennifer Town, Nina Cui, Patrice Audy, Sue Boyetchko, Tim J. Dumonceaux

**Affiliations:** a Agriculture and Agri-Food Canada, Saskatoon Research and Development Centre, Saskatoon, Saskatchewan, Canada; b Department of Veterinary Microbiology, University of Saskatchewan, Saskatoon, Saskatchewan, Canada; c Department of Biology, University of Waterloo, Waterloo, Ontario, Canada; d Agriculture and Agri-Food Canada Québec Research and Development Centre, Hochelaga, Québec, Canada

## Abstract

*Pseudomonas* sp. strain KENGFT3 inhibits the growth of *Phytophthora infestans* and is a potentially useful biopesticide for plant diseases, including potato late blight. We sequenced the 6.2-Mbp genome of this strain and assembled it into a single scaffold with 9 contigs. KENGFT3 is related to previously sequenced strains of *P. fluorescens*.

## GENOME ANNOUNCEMENT

*Pseudomonas* sp. strain KENGFT3 is a Gram-negative bacterium isolated from soil near Kenaston, Saskatchewan, Canada. This organism inhibits the growth of the potato pathogen *Phytophthora infestans* in *in vivo* challenge assays and has been identified as a potential biocontrol agent for potato late blight.

*Pseudomonas* sp. KENGFT3 was grown at 22°C in a rotary shaker for 24 to 48 h in yeast extract glucose medium (2.0 g/liter yeast extract, 2.5 g/liter glucose, 0.4 mM MgSO_4_·7H_2_O, 0.09 mM MnSO_4_·H_2_O, 0.85 mM NaCl, 0.017 mM FeSO_4_·7H_2_O, 1.84 mM KH_2_PO_4_, and 1.43 mM K_2_HPO_4_). Genomic DNA was purified from 1 ml of overnight culture using the Wizard gDNA extraction kit (Promega, Madison, WI, USA) and sequenced on the GS Junior using Titanium Plus chemistry (Roche Diagnostics, Laval, Quebec, Canada). Reads from two shotgun sequencing runs with average read lengths of 548 and 534 bp were assembled using Newbler version 3.0 (454 Life Sciences). The total number of filter-passed reads was 128,460, and the total number of bases assembled was 69,421,957. These reads were assembled into 97 large contigs, with an *N*_50_ contig size of 131,172 kbp. In addition, an 8-kb-insert paired-end sequencing run was performed based on the paired-end rapid library preparation protocol for Titanium chemistry (Roche), with modifications as described (1). A total of 105,459 paired-end reads were generated, with an estimated pair distance of 6,361 ± 1,590 bp. Assembly of all the sequencing runs together produced an improved high-quality draft sequence (2) featuring 16× genome coverage of a single scaffold with 9 scaffold contigs. Sequence data were annotated using the Prokaryotic Genome Annotation Pipeline version 2.0 (NCBI).

The genome of *Pseudomonas* sp. KENGFT3 contained 6,183,312 bp (59.95% G+C content). A total of 5,791 genes and 5,549 protein-coding genes were observed, along with 6 genes encoding 5S rRNA, 5 genes encoding 16S rRNA, 5 genes encoding 23S rRNA, and 64 tRNA-coding genes. The majority (77.76%) of protein-coding genes had a predicted function, and 2,053 COG clusters were identified.

SpecI (3) could not assign *Pseudomonas* sp. KENGFT3 to a species cluster (the average nucleotide identity was 95.1% to *Pseudomonas fluorescens* strain SBW25; GenBank accession no. NC_012660.1). JSpecies (4) also revealed that *Pseudomonas* sp. KENGFT3 had genome comparison metrics that placed it below the identified cutoff for inclusion in the same species as *P. fluorescens* SBW25. However, *P. fluorescens* strain LBUM223 (5), which shares certain phenotypic attributes with KENGFT3 (6, 7), had genome comparison metrics that placed these two strains within the same species (average nucleotide identity [ANI], 99.39%). Calculation of a phylogenetic distance tree using 60 strains of *P. fluorescens* annotated at the Integrated Microbial Genomes portal (https://img.jgi.doe.gov/cgi-bin/mer/main.cgi) revealed that *P. fluorescens* KENGFT3 and LBUM223 clustered with *P. fluorescens* strains GcM5-1A and UK4. *P. fluorescens* KENGFT3 possessed an array of genes that have been associated with biocontrol phenotypes, including phenazine carboxylic acid synthesis (8), chitinases and cellulases (9), and pyrroloquinoline quinone biosynthesis (10), among others. Ten genes encoding putative β-lactamases were also found.

### Nucleotide sequence accession number.

The sequence data for this complete genome have been deposited at DDBJ/EMBL/GenBank under the accession no. CP014868.
